# Recent progress on Tourette syndrome

**DOI:** 10.12703/r/10-70

**Published:** 2021-09-07

**Authors:** Keisuke Ueda, Kevin J Black

**Affiliations:** 1 Department of Neurology, Washington University School of Medicine, St. Louis, MO, USA; 2 Department of Psychiatry, Washington University School of Medicine, St Louis, MO, USA; 3 Department of Radiology, Washington University School of Medicine, St Louis, MO, USA; 4 Department of Neuroscience, Washington University School of Medicine, St Louis, MO, USA

**Keywords:** Tourette syndrome, Tics, Tic disorders, Neuropsychology, Comorbidity

## Abstract

Tic disorders and Tourette syndrome are the most common movement disorders in children and are characterized by movements or vocalizations. Clinically, Tourette syndrome is frequently associated with comorbid psychiatric symptoms. Although dysfunction of cortical–striatal–thalamic–cortical circuits with aberrant neurotransmitter function has been considered the proximate cause of tics, the mechanism underlying this association is unclear. Recently, many studies have been conducted to elucidate the epidemiology, clinical course, comorbid symptoms, and pathophysiology of tic disorders by using laboratory studies, neuroimaging, electrophysiological testing, environmental exposure, and genetic testing. In addition, many researchers have focused on treatment for tics, including behavioral therapy, pharmacological treatment, and surgical treatment. Here, we provide an overview of recent progress on Tourette syndrome.

## Introduction

**Figure 1.**
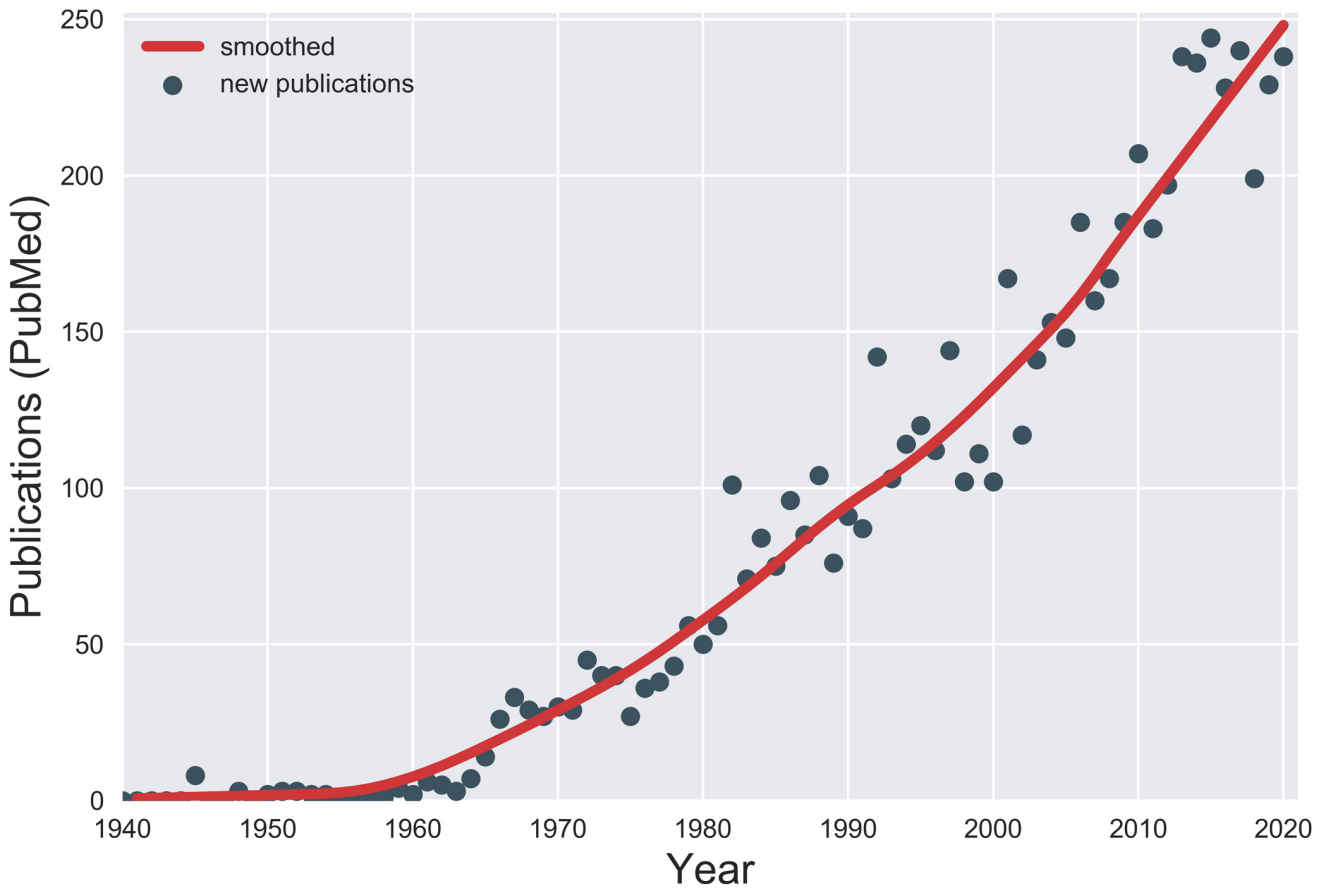
Publications on Tourette syndrome. The number of new publications on Tourette syndrome or other tic disorders each year was estimated from PubMed. PubMed was searched by using the search string “(“Tic Disorders”[MeSH] OR Tourette NOT Tourette[AU]) AND *year*[PDAT] NOT *year+1*[PDAT]” for each year from 1940 through 2020.

Tic disorders are characterized by sudden, rapid, recurrent movement (motor tics) or vocalization (vocal or phonic tics)^1^. Tourette syndrome (TS) indicates the presence of multiple motor and vocal tics spanning a period of more than 1 year, and onset is before the age of 18 years. The cardinal features of tics are a premonitory urge (an unpleasant sensation preceding tics)^2^, suppressibility (the ability to voluntarily suppress tics for variable periods)^3^, and suggestibility (more likely to experience a tic when it is mentioned)^4^. Tics can be associated with various psychiatric comorbidities, which can complicate the clinical picture. Since the most recent previous review in this journal^5^, the number of publications on tic disorders has been steadily increasing (Figure 1), leading to a need for this update^6^. Thus, we have reviewed recent progress on the clinical course, epidemiology, comorbidities, pathophysiology, and treatment of TS. We searched PubMed for articles published between 2018 and 2021 and used search terms such as tic disorders or Tourette. We then subjectively selected articles that met the objectives of this review. We also included articles that were recommended by colleagues.

## Clinical course and phenomenology

Recent years have seen changes to definitions of tic disorders. The *Diagnostic and Statistical Manual of Mental Disorders, 4th edition, Text Revision* (DSM-IV-TR) required a year of tics, excluding any tic-free period of three months or longer, for the diagnosis of TS; however, the three-month requirement was removed in the DSM-5^1^. The International Classification of Diseases 10th revision (ICD-10) required “multiple motor tics” and one or more vocal tics for the diagnosis of TS, but the requirement for more than one motor tic was removed in ICD-11^7^.

Tic symptoms generally follow a fluctuating course in terms of severity and frequency, with a mixture of old and new tics, and may be exacerbated by psychological and physical strains (such as anxiety or fatigue) and environmental changes^8,9^. Traditionally, experts have asserted that a tic disorder that began only in the past few months would usually disappear within a year; however, a direct follow-up study revealed that at least 90% of children still exhibited tics after a year^10^. Among them, children who could suppress tics better under conditions of immediate reward showed less severe tics at follow-up^11^. A population-based longitudinal study of parents and children with TS showed that low socioeconomic status was a risk factor for TS and chronic tic disorders^12^. A longitudinal cohort study of children with TS at the Danish National Tourette Clinic identified that severity of tics, attention-deficit/hyperactivity disorder (ADHD), and obsessive–compulsive disorder (OCD) in childhood strongly predicted the corresponding severity scores in early adulthood^13^. In the same group, 18% of participants who were at least 16 years old were tic-free, 60% had minimal or mild tics, and 22% had moderate to severe tics, indicating an age-related decrease in tics^14^.

The Yale Global Tic Severity Scale (YGTSS) was invented to rate tic severity^15^. A large cross-sectional study of 617 adults and children with tic disorders found good internal consistency of the YGTSS between children and adults but suggested some modifications to improve its usability and value^16^. Similarly, the European Multicentre Tics in Children Study (EMTICS), which includes 16 clinical sites, examined the psychometric quality of the YGTSS and found overall good results but suggested some modifications, including that the total tic severity scale and the impairment rating be used separately^17^.

Both motor and vocal tics are categorized as simple or complex^18^. Tics can be further classified as clonic, tonic, or dystonic on the basis of their phenomenology^19,20^. Clonic tics are abrupt, brief jerking movements. Dystonic tics are sustained abnormal movements, whereas tonic tics are isometric contractions. Some authors add blocking tics, namely transient interruptions of ongoing motor activities or speech without loss of consciousness^20^. A prospective study identified tonic tics in 85% of adults and 64% of children with TS; tonic tics correlated with the number of tics, severity, and comorbidities^21^.

Functional tic-like movements, which are usually rare in clinical practice, have recently drawn attention. Clinical features of tics and functional tic-like movements overlap and can coexist, which can make it difficult to differentiate tics from functional tic-like movements^22^. Compared with TS, functional tics are thought to be characterized by lack of premonitory urge and suppressibility, female preponderance, the presence of other functional symptoms, resistance to tic medications, and lack of positive family history of tic disorders^22^. During the Covid-19 pandemic, we encountered many adolescents with sudden onset of severe tics and tic-like movements. Some of the cases may be explained by increased stress exacerbating pre-existing tic disorders in the context of widespread social stressors, but other cases were more consistent with a functional tic-like disorder^23^. Some patients before onset of their symptoms had watched videos of social media influencers demonstrating similar tic symptoms, suggesting probable social contagion^23^.

## Epidemiology

The true prevalence of tic disorders is difficult to estimate owing in part to symptom fluctuation over time and heterogeneous presentations^24^. In a meta-analysis of school-based studies, the prevalences of transient tic disorder (now called provisional tic disorder, tics of less than 1 year), chronic vocal tics (phonic tics of more than 1 year but no motor tics), chronic motor tics (motor tics of more than 1 year but no phonic tics), and tic disorder not otherwise specified (tics that do not meet criteria for other tic disorders) were estimated to be 3.0%, 0.7%, 1.7%, and 0.8%, respectively^25^. However, this analysis excluded several studies that used direct observation to identify tics, and these studies generally reported much higher prevalence. A meta-analysis using data from population-based studies conducted in China reported overall prevalences of 1.7% for transient tic disorder and 1.2% for chronic motor or vocal tic disorders^26^. A systematic review of 21 population-based studies of children who were 4 to 18 years old estimated the prevalence of TS to be 0.52% (95% confidence interval [CI] 0.32‒0.85)^27^, although some high-quality studies find prevalence to be as much as 10-fold higher^28^. In contrast, prevalence is much lower in adults; a meta-analysis of three studies involving 2,356,485 adults estimated the prevalence to be 0.001% (118 cases per million)^29^. However, given that most children with recent onset of tics continue to have tics for over 1 year^10^, this result probably underestimates the true prevalence of TS among the general population. A possible explanation for this is that a substantial number of patients with tics do not seek medical attention or do not realize they have tics since their tics are subtle and not troublesome. Therefore, a large population-based observational study is needed to evaluate the true prevalence of TS.

## Pathophysiology

The pathophysiology of tic disorders is complicated, and studies have implicated cortical–striatal–thalamic–cortical (CSTC) circuits and associated neurotransmitters^30,31^. Various methods, including neuroimaging, physiological, laboratory, animal, and genetic studies, have been used to unveil its pathophysiology.

### Neuroimaging studies

Several neuroimaging studies have investigated anatomical changes as well as structural and functional connectivity^32^. A follow-up study using high-resolution magnetic resonance imaging (MRI) in 41 children with new-onset tics showed that larger hippocampal volume at the baseline visit predicted higher tic severity at 1-year follow-up^33^. A high-resolution MRI study using voxel-based cerebellar morphometry and seed-to-voxel structural covariance mapping (structural connectivity) demonstrated reduced cerebellar gray matter volume in patients with TS, which was considered to affect higher-order cognitive and motor processing^34^. The study also showed abnormal gray matter structural connectivity with frontal and cingulate cortices and sensorimotor networks, indicating that cerebellar involvement in tics is part of cortico–basal ganglia–cerebellar interactions.

Functional imaging studies have been providing new insights into the temporal correlation of activity between different brain regions (i.e., functional connectivity)^32^. To investigate changes of functional brain organization in TS with development, a resting-state functional connectivity MRI study of 172 children and adults with TS was performed^35^. Functional connectivity alteration was age-specific: brain networks in children with TS appeared “older” whereas brain networks in adults with TS appeared “younger” than those in age-matched controls^35^. A resting-state functional MRI (fMRI) study using graph theoretical measures focused on topological changes of basal ganglia–thalamocortical and cortico–cerebellar brain networks in TS^36^. Patients with TS showed increased basal ganglia–cortical and thalamo–cortical connectivity and reduced cortico–cerebellar connectivity. They also showed decreased connectivity within the precuneus, posterior cingulate, and right and left angular gyri and within the right and left insular cortex. The altered connectivity may suggest a lack of brain maturation and interoception involvement as neural correlates of tics and premonitory urges.

A functional imaging study using whole-brain structural MRI with voxel-based morphometry techniques and seed-to-voxel structural covariance mapping was conducted to investigate structural connectivity of tic severity and of premonitory urges^37^. Motor severity and premonitory urge severity were not correlated and were associated with anatomically separate regions of the right insular cortex. This result suggests that tics and premonitory urges have separate network connectivity. A resting-state fMRI with seed-based functional connectivity analysis was conducted to investigate neuronal functional connectivity of tics, OCD, and premonitory urge^38^. Greater connectivity between the putamen and sensorimotor cortex was noted in participants with TS, whereas less connectivity between the supplementary motor area (SMA) and thalamus and between the caudate and precuneus was noted in participants with obsessive–compulsive symptoms. Moreover, a case-control study using diffusion tensor imaging MRI and transcranial magnetic stimulation (TMS) over the right pre-SMA was conducted in children with chronic motor tics and healthy controls^39^. The study showed that pre-SMA-mediated motor cortex inhibition was impaired in participants with tic disorders; less inhibition correlated with worse tic suppressibility and more severe tics^39^. The functional connectivity within sensorimotor regions might play a role in tic severity, premonitory urges, suppressibility, and obsessive–compulsive symptoms.

### Electrophysiology studies

Electrophysiological methods are well suited to studying temporal brain dynamics. A narrative review summarized that electrophysiological studies, including electroencephalogram (EEG) and event-related potential studies, could echo neuronal processes and parallel clinical characteristics of TS^40^. A case-control study using resting-state EEG of patients with TS showed decreased frontotemporal–occipital–parietal connectivity and a dysfunctional network of intrinsic long-range connectivity between the frontal and temporal–occipital–parietal lobes and proposed these as potential biomarkers for TS^41^. Local field potentials can be recorded during deep brain stimulation (DBS) implantation surgery. A retrospective clinical study of 17 patients with TS who underwent DBS of the thalamus showed that increased power in the low-frequency (5–15 Hz) band could be a biomarker for TS and correlated with the severity of tics^42^.

### Laboratory studies

Recently, new evidence has supported dopaminergic involvement in the pathophysiology of TS. A case-control study measuring urine tetrahydroisoquinolines, which modulate dopaminergic neurotransmission and metabolism in the central nervous system, demonstrated a significant increase in the levels of norsalsolinol in patients with TS and those with TS and ADHD, suggesting dopaminergic hyperactivity in the pathophysiology of TS^43^. Another case-control study measuring cerebrospinal ﬂuid (CSF) levels of endocannabinoids demonstrated significant alterations in the levels, suggesting an involvement of the endocannabinoid system in the pathophysiology of TS^44^. The study authors speculated that an increased level of endocannabinoids might be a compensation for dopaminergic hyperactivity.

Animal studies have also suggested CSTC circuit involvement in TS pathophysiology. Postsynaptic SAP90/PSD95-associated protein 3 (SAPAP3) is highly expressed in the striatum and plays an important role in cortico-striatal circuitry in OCD-like behaviors, as shown in a mouse experiment^45^. Sapap3-knockout mice exhibited tic-like movements with short, sudden, repetitive movements (e.g., rapid head twitching and body twitching)^46^. The tic-like movements in Sapap3-knockout mice were reduced significantly by treatment with aripiprazole, an atypical antipsychotic drug used to treat tics^46^. Another animal experiment was conducted using D1CT-7 transgenic TS mice^47^. D1CT-7 mice are a “transgenic line generated through the attachment of a neuropotentiating cholera toxin to the D1 receptor promoter” and have been considered a good animal model of TS^48^. The researchers administered DK-I-56-1, a selective positive allosteric modulator of GABA-A receptor containing alpha6 subunits, to D1CT-7 transgenic TS model mice and found that DK-I-56-1 was as effective as D1 and D2 receptor antagonists and significantly reduced tic-like movements^47^. MicroRNAs (miRNAs) have attracted attention as biomarkers for diseases, intercellular communication, and neural development^49^. A clinical study using serum miRNA expression proﬁles as molecular fingerprints for children with either TS or Arnold–Chiari malformation showed nine differentially expressed miRNAs as potential molecular tools for the diagnosis of TS^50^. These pathophysiological studies have converged to suggest the importance of CSTC circuits and their associated neurotransmitters and other brain regions but have not yet supported a clear, inclusive theory of tic pathophysiology.

## Etiology

### Environmental factors

Environmental influences such as prenatal and perinatal epigenetic factors and inflammatory factors have been investigated as underlying causes of tic disorders. A systematic meta-analysis found a 35% increase in the risk of tic disorders in offspring with maternal smoking during pregnancy (pooled relative risk 1.35, 95% CI 1.17‒1.56)^51^. Similarly, a prospective case-control study showed that maternal pro-inflammatory states were associated with tics and OCD in children, supporting a possible role of maternal inflammation^52^.

Inflammation has been extensively discussed in tic pathophysiology. A recent review article on immunological mechanisms in the pathophysiology of tic disorders argues that innate and adaptive systemic immune pathways and neuroinflammatory mechanisms play an important role in the pathogenesis of at least some patients with TS^53^. The article also postulated that hyper-reactive systemic immune pathways and neuroinflammation may help explain the natural fluctuations of tic disorder symptoms over time.

The prevalence and characteristics of tics in patients with encephalitis were reviewed in a systematic study, which found that sporadic cases of tics were associated with encephalitis, particularly during a post-encephalitis period, and with basal ganglia involvement^54^. A case-control autopsy study (of nine individuals with TS) using basal ganglia transcriptome by RNA sequencing in the caudate and putamen found disrupted basal ganglia neuronal signaling^55^. The study also found a significant increase in immune and inflammatory transcripts. These results suggest metabolic alterations and inflammatory involvement in TS pathophysiology. A comprehensive review of immune dysfunction in TS discussed clinical correlation with group A Streptococcus (GAS) infection, autoantibody analysis, and gene expression studies and identified some support for a hypothesis of autoimmune dysfunction as the pathophysiology of TS and its associated neuropsychiatric symptoms^56^.

CSF analyses have revealed inflammatory mechanisms in tic disorders. The longitudinal EMTICS study investigated the role of environmental exposure and immunology in the clinical course and comorbidities of TS^57^, and immunohistochemistry staining on rat brain sections failed to show evidence of specific neuronal surface antibodies (such as NMDA, CASPR2, LGI1, AMPAR, and GABAAR) in children with TS^58^. A prospective CSF analysis study demonstrated positive oligoclonal bands in 20% of participants (4 out of 20) but did not compare that rate with that of tic-free control participants and observed no specific surface autoantibodies, such as NMDA, CASPR2, LGI1, AMPA, and GABA1/B, and no specific binding pattern^59^. Although specific surface antibodies were not detected, the presence of oligoclonal bands in CSF analysis could suggest an autoimmune etiology in TS^59^. The authors suggested a pathological immune process of intrathecal IgG antibody production, considering that other studies find OCB positive in only 5% of healthy individuals and it is rarely seen in patients with non-inflammatory diseases.

On the other hand, the EMTICS study also examined whether exacerbations of tics and comorbid symptoms were associated with GAS exposure, which was determined by throat swabs and serum antibodies^60^. GAS exposure was not significantly associated with tic exacerbations (odds ratio 1.006‒1.235; *P* >0.3); however, it was associated with hyperactivity–impulsivity symptoms. The study authors concluded that GAS exposure was less likely to be a risk factor for tic exacerbations; therefore, the evaluation and treatment of GAS infections are not warranted in the context of worsening of tics. The authors also attempted to identify biomarkers of tics, examining hair cortisol concentration as a physiological marker of long-term stress in patients with tics, and found no association between hair cortisol concentration and tic severity^61^.

### Genetics

Tic disorders are polygenic inherited disorders involving different genes. In a population-based cohort study in Sweden using the Genome-wide Complex Trait Analysis program, the heritability estimate of TS was 0.58 to 0.77, and the odds ratio for tic disorders of first-degree relatives of probands with tic disorders (18.7) was significantly higher than that of the second-degree (4.6) and third-degree (3.1) relatives^62,63^.

Several candidate susceptibility genes for TS have been identified but have not been confirmed and this is likely due to the small sample size of each study and genetic and phenotypic heterogeneity^64^. The *SLITRK1* gene on chromosome 13q31.1 encoding a single-pass transmembrane protein in the central nervous system was reported to be associated with TS^65^. The *IMMP2L* gene encoding the inner mitochondrial membrane peptidase subunit 2 was implicated in TS^66^, but a recent study using skin fibroblasts from adults with the *IMMP2L* deletions and TS failed to show evidence of mitochondrial dysfunction^67^. Expression analysis, genotyping, and methylation analysis of *SLC6A4* in 57 patients with TS showed a significantly higher expression of *SLC6A4* mRNA, which encodes serotonin transporter, compared with healthy controls^68^. *SLC6A4* overexpression may contribute to TS pathophysiology by increasing serotonin clearance. Whole-exome sequencing studies identified the *CELSR3* gene on chromosome 3p21.31^69^, the *ASH1L* gene on chromosome 1q22^70^, and possibly disrupted variants of the *OPRK1* gene on chromosome 8q11.23, encoding the opioid kappa receptor^71^ as high-risk genes for TS.

A study of 802 TS trios found that novel mutations were more common in simplex but not multiplex families and identified a greater-than-expected number of mutations related to cell polarity^69^. That study also identified shared genetic variance with OCD and autism spectrum disorder. The Brainstorm Consortium Genome-wide association study data on 3581 patients with TS and 7682 controls identified three significant gene sets involving ligand-gated ion channel signaling, lymphocytic and cell adhesion, and trans-synaptic signaling processes^72^.

## Comorbidities

TS is frequently accompanied by ADHD, OCD, anxiety, depression, autism spectrum disorder, sleep disorders, migraine, rage attacks, and self-injurious behavior (SIB)^73–77^. Aggressive behavior measured by the Overt Aggression Scale is associated with the severity of comorbid ADHD^78^. According to a systematic literature review, 35% of patients with TS had SIB, and obsessive–compulsive behaviors were correlated with SIB in patients with TS^74^. Similarly, a cohort study of Polish patients with TS showed that SIB was associated with tic severity, OCD, and ADHD^79^. To understand the prevalence of psychiatric comorbidities, a cross-sectional interview study of 1374 adolescents and adults with TS and 1142 TS-unaffected family members was conducted. The study found that 86% of patients with TS had a lifetime prevalence of any psychiatric symptoms (excluding tics) and 58% of them had more than two psychiatric illnesses^80^. The severity of tics as well as symptoms of the two most common comorbidities—ADHD and OCD—declined during adolescence in a 6-year Danish cohort study of 314 children and adolescents with TS^14^.

Non-psychiatric comorbidities have been increasingly reported. A study using data from the National Health Insurance Research Database of Taiwan showed an increased risk of traumatic brain injury (TBI) in patients with previously diagnosed TS (hazard ratio [HR] 1.59, 95% CI 1.37–1.85)^81^. Moreover, patients with TS undergoing antipsychotic treatment had a lower risk of TBI than infrequent users (HR 0.76, 95% CI 0.57–0.99). The study authors speculated that the improvement of tics with antipsychotics protected against TBI or that antipsychotics reduced their impulsivity (or SIB). A large population-based cohort study using the Swedish National Patient Register has shown that individuals with tic disorders have a higher risk of transportation-related injuries and death than the general population (adjusted HR 1.50, 95% CI 1.33‒1.69); however, TS individuals without ADHD did not have a significantly elevated risk, suggesting that comorbid ADHD was the contributor to the automotive injuries^62,82^. The same group showed a higher risk of metabolic and cardiovascular disorders such as obesity (adjusted HR 2.76, 95% CI 2.47‒3.09), type 2 diabetes (adjusted HR 1.67, 95% CI 1.42‒1.96), and circulatory system diseases (adjusted HR 1.76, 95% CI 1.67‒1.86) in patients with TS compared with the general population^83^. Surprisingly, use of antipsychotics for more than 1 year significantly *decreased* the risk of metabolic and cardiovascular disorders^83^. The risk of substance misuse (alcohol, drugs, and substance-related crimes) in individuals with TS was higher than that in the general population (adjusted HR 3.11, 95% CI 2.94‒3.29)^84^.

Although it is generally assumed that most patients with tic disorders have normal intelligence, the association between TS and cognition is unclear^85^. A case-control study on learning ability in TS revealed impairment of visual associative learning (a style of learning where one learns things by associating them with a stimulus), but retrieval and generalization were not affected^86^. Another case-control study using an automatic imitation task showed that participants with TS responded faster than controls but had higher error rates, suggesting a different control mechanism of their motor responses to sensory stimuli from observed actions^87^. Yet another case-control study examined the executive function and psychomotor speed of TS children with ADHD, TS children without ADHD, ADHD children without tics, and controls and showed that the severity of ADHD possibly affects executive dysfunction^88^. ADHD appears to play a role in learning, and ADHD treatment may improve learning and executive function, but more research is necessary. A case-control study in Sweden showed that treatment-seeking individuals with tic disorders experienced academic underachievement from primary school through university^89^.

Owing to heterogeneous symptoms, several groups have attempted to categorize or view tic disorders and TS as a spectrum. Using a cluster dendrogram of hierarchical ascendant clustering based on comorbidities, a prospective clinical study of 174 patients with TS demonstrated three clusters: TS with no other neurodevelopmental comorbidities; TS with higher intelligence, attention deficit, and handwriting problems; and TS with neurodevelopmental comorbidities, learning disabilities, and academic impairments^90^. A retrospective analysis of 1018 patients with TS and chronic motor tic disorders (motor tics for more than 1 year but no phonic tics) found that TS and chronic tic disorders were not distinct in clinical or demographic variables and suggested that “tic spectrum disorders” with TS were more severe and were associated with more comorbidities whereas chronic motor tics were less severe^91^. A very compelling data analysis and review concludes that TS and chronic motor or phonic tic disorder are the same illness or at least that they occur on a spectrum with TS at the more severe end^92^. In clinic settings, the term TS can occasionally provoke avoidance from patients and their guardians because of myths and misconceptions created by social media. These findings will allow clinicians to advise patients on the expected clinical course of tics and other comorbidities, and the concept of “tic spectrum disorder” may help patients and guardians accept the diagnosis.

Although a large interview study across nine academic TS and OCD specialty clinics showed a correlation between tic severity and tic impairment^93^, non-tic-related symptoms can be more problematic than tics themselves^94^. Paying attention to comorbid symptoms and treatment is often more important than addressing tics themselves. Continual assessment of comorbidities by using a scale or assessment tool is warranted. For example, the mini-child Tourette syndrome impairment scale, a parent- and child-reported tic impairment scale, was invented to quantify tic-related and non-tic-related impairment across the school, home, and social domains and was shown to be well correlated with tic and comorbid symptom severity^95,96^.

## Treatment

The European Society for the Study of Tourette Syndrome (ESSTS) is about to release an updated version of its 2011 guidelines for TS, and the revised guidelines will cover in detail many of the points we touch on in this section^97^.

### Behavior therapy

Behavioral therapies are recommended as the first-line treatment for tics by the American Academy of Neurology practice guidelines^98^. These therapies consist of exposure and response prevention (ERP), habit reversal therapy (HRT), or its descendant comprehensive behavioral interventions for tics (CBIT)^99^. A randomized control trial evaluating the long-term effect of both HRT and ERP in children and adults with tic disorders showed that the benefit of the therapies persisted at the 1-year follow-up visit in 74% of participants^100^. Despite robust evidence of the behavioral therapies to reduce tic frequency and severity and diminish the urges, finding a trained therapist can be challenging. To improve access to the therapies, internet-based training programs, which have shown improvement in parent-rated tic severity, tic-related impairment, and quality of life, have been developed^101,102^. Group-based behavioral therapies have also been investigated because group therapies are more cost-effective, treating more patients at once. They can also provide an opportunity for patients to meet in person, share their experiences, and support each other. An open-label controlled clinical trial of combined HRT and ERP was conducted in adolescents with TS^103^. The participants were randomly assigned to either individual or group therapy. In total, 67% of participants were considered responders, and there was no significant difference between individual and group therapies. Furthermore, group-based CBIT for children and adolescents showed a significant reduction in tics and severity of comorbid symptoms such as anxiety, behavioral problems, and aggressive behaviors^104,105^.

### Pharmacological therapy

Pharmacological treatment is considered when behavioral interventions fail or are unavailable or when urgent benefit is required. Various medicines, including alpha-2 adrenergic agonists, antiepileptic drugs, and dopamine receptor blocking agents, are commonly used for the treatment of tics^98^. Most patients with tics respond to behavioral therapy or tic-suppressing medications. However, some do not, and others experience problematic side effects, so new treatment modalities are being investigated. Recently, novel drugs have been investigated for the treatment of tics. Tiapride, a dopamine receptor blocking agent, is used mainly in Europe for the treatment of tics. A clinical study of children with TS showed that 83% of participants responded to tiapride and no patients had serious adverse reactions^106^. Ecopipam, a selective D1 receptor agonist, was studied in a randomized, placebo-controlled crossover study of children and adolescents with TS^107^. Ecopipam significantly reduced tic severity at 16 days (95% CI −6.5 to −0.9; *P* = 0.011) and 30 days (95% CI −6.1 to −0.3; *P* = 0.033) and was well tolerated without serious side effects. Lurasidone, a dopamine and serotonin receptor antagonist, is an atypical antipsychotic used for the treatment of schizophrenia and bipolar disorder^108^. Six children and adolescents with treatment-refractory TS, aggressive behavior, and obsessive symptoms responded significantly to lurasidone as an add-on therapy to risperidone or aripiprazole^109^.

Dopamine-depleting agents block the vesicular monoamine transporter type 2 and are used to treat hyperkinetic movement disorders such as chorea, tardive dyskinesia, and tics^110^. Tetrabenazine, a dopamine-depleting agent, was found to be effective in an open-label study of 120 patients with tics^111^. Subsequently, an open-label study of 28 children and adolescents with TS investigated valbenazine, which is a purified parent drug of the (+)-α-isomer of tetrabenazine, but it failed to show statistically significant efficacy^112^. A trial of deutetrabenazine, a deuterated form of tetrabenazine, also failed to show a significant benefit (ARTISTS1).

The use of cannabis and cannabis-derived products has been reported to improve tics. A study of TS model rodents demonstrated that delta-9-tetrahydrocannabinol might reduce tic behaviors and premonitory urges in young adult mice but might also exacerbate tic behaviors in juvenile mice^113^. A retrospective data analysis of adults with TS who received cannabis-based medicine showed a subjective improvement in tics, comorbidities, and quality of life, although adverse effects were noted in half the patients^114^. A randomized clinical trial to evaluate the safety and efficacy of cannabis in adults with TS was launched in 2018, but it was terminated because the recruitment and enrollment were prolonged (ClinicalTrials.gov Identifier: NCT03247244). A multicenter, randomized, double-blind, placebo-controlled trial of patients with tic disorders is being conducted to investigate the use of the cannabis extract nabiximols for the treatment of tics^115^.

Complementary and alternative medicines—including dietary or nutritional supplements (calcium, magnesium, coenzyme Q_10_, fish oil, gastrodin, and vitamins B, C, D, and E), chiropractic manipulations, meditation, acupuncture, hypnosis, homeopathy, and biofeedback—have been reported for the treatment of tics^116,117^; however, the evidence is limited because of a lack of randomized control studies. The efﬁcacy and safety of a Chinese herbal medicine (5-Ling granule) in the treatment of TS were evaluated in a multicenter, double-blind randomized controlled trial, finding it as effective as tiapride in improving tic symptoms^118^.

TMS is a non-invasive brain stimulation treatment to modulate neural plasticity and has been attracting considerable attention^119^. An open-label clinical trial (of 10 children with TS) using low-frequency repetitive TMS to the bilateral SMA for 15 sessions showed a statistically significant decrease in tic severity^120^. These results are promising but a large randomized trial is warranted to validate the findings.

### Surgical therapy

DBS may be a promising neurosurgical treatment for tics^121^. Patient selection criteria and an algorithm for DBS treatment for TS, consisting of five pillars, have been proposed: high tic severity, tic-related impact on quality of life, failure of behavioral and pharmacological treatment, stability of comorbid symptoms, and age of 18 years or more^122^. The International Deep Brain Stimulation Database and Registry reports an overall adverse event rate of 35%, and medically serious complications—intracranial hemorrhage (1.3%), infection (3.2%), and lead explantation (0.6%)—were less common than stimulation-induced adverse effects, including dysarthria (6.3%) and paresthesia (8.2%)^121^. Tic-like behaviors have also been reported as adverse effects of DBS. A patient who underwent DBS of the ventral internal capsule and ventral striatum for treatment-resistant major depressive disorder developed stimulation-dependent TS-like behaviors such as motor tic-like movements in the arms as well as coprolalia and stuttered speech^123^.

There have been many reports of patients with TS who underwent DBS in various targets^121^. A double-blind randomized controlled trial of bilateral anterior globus pallidus pars interna (GPi) DBS was conducted in 19 patients with TS; one group received active stimulation and the other group received sham stimulation^124^. No significant difference in tic severity was noted three months after surgery. However, in an open-label, follow-up study, 75% of patients had an average 70% tic reduction in the YGTSS total score after 48 months^125^. Posteroventral GPi DBS has also been reported^126^. In addition to the GPi, the thalamus, globus pallidus externus, anterior limb of the internal capsule, and nucleus accumbens have been suggested as targets for DBS for tics^127^. An adult with TS who underwent bilateral dual-targeted DBS to the centromedian–parafascicular complex (CM-Pf) and the ventral capsule/ventral striatum reported benefits in terms of motor and non-motor TS symptoms^128^. A prospective clinical study of 25 patients with TS who underwent DBS to the CM-Pf showed improvement of tics by 45% at the 1-year follow-up^129^. Another, retrospective, study focused on DBS with a target of either the ventralis oralis (Voi) or the CM-Pf of the thalamus (41 patients) and the anteromedial GPi (am-GPi) (14 patients)^130^. They found possible superiority of the am-GPi to the Voi/CM-Pf for the treatment of obsessive–compulsive symptoms in TS; however, target selection was not random, as the authors note the CM-Pf was preferred for patients with impairment mostly from tics rather than comorbidities. The International Tourette Syndrome DBS Database and Registry conducted a retrospective, probabilistic tractography study of DBS targeting the GPi or CM^131^. Stimulus-dependent connectivity to specific regions was shown to be likely to mediate improvement in tics^131^. Specifically, the improvement of tics by DBS in GPi can be attributed to modulation of the limbic and associative networks by optimally positioned stimulation contacts, while the improvement of tics by DBS in the medial thalamus can be attributed to modulation of the sensorimotor and parietal–temporal–occipital networks. This finding could be used to refine the neuromodulation targets and stimulation parameters for tic disorders. Currently, it is common to select targets on the basis of clinical symptoms. However, considerable debate remains about the optimal DBS target (or targets) in TS, and there are no evidence-based guidelines to assist in the selection of DBS target in TS. Most studies that reported successful DBS results for the treatment of TS are case reports or open-label studies with various targets, and randomized control studies have shown inconsistent results^124,132–134^. Some investigators have interpreted the greater benefit in open-label trials as evidence of our difficulty predicting ideal lead location and pulse characteristics for treating tics. In fact, considerable debate remains about the optimal DBS target (or targets) in TS, and there are no evidence-based guidelines to assist in the selection of DBS target in TS. However, in other movement disorders, the stringent requirements of randomized controlled trials have not prevented DBS from producing relatively dramatic results (e.g., in dystonia^135^ or Parkinson disease^136^). Alternatively, the better results in open-label studies may indicate expectation or placebo effects, even in patients with treatment-resistant TS treated with DBS. Placebo benefit can be demonstrated directly in DBS for Parkinson disease^137^. Thus, further studies are needed to identify the optimum target, benefits, risks, and stimulation parameters for DBS in TS^138^, and in our current state of knowledge, selection of patients for DBS surgery, including patients with severe TS, should be done quite cautiously^122,139^.

## Conclusions

Although tics are a common movement disorder in children, there are still many unanswered questions, including on the causes and natural history of tics and their relationship with comorbid symptoms. Increasingly many research studies are published each year and have provided new insights into the pathophysiology and etiology of tics. Recent years have seen increasing information about the relationship between tics and comorbidities and about new genetic findings. Inflammatory processes have also been a topic of continued interest.

We believe the near future for Tourette research may include more large-scale collaborative studies, which can provide more powerful results. Advances in imaging technology are also likely to enhance our understanding of tics. Treatment of TS is becoming more standardized with the recent American Academy of Neurology and forthcoming ESSTS guidelines. Behavioral therapy interventions are becoming more widely accepted but still have limitations on availability. However, alternative approaches, including internet-delivered and group therapy, are being explored. Furthermore, new medications are still needed and likely will lead to improved treatments. Substantial debate continues about DBS therapy, its effectiveness, and optimal targets, and the international DBS registry will continue to move the field forward. We remain optimistic about improved care for TS that future research in all these areas is likely to produce.
